# The association between interpersonal problems and treatment outcome in patients with eating disorders

**DOI:** 10.1186/s40337-017-0179-6

**Published:** 2017-11-21

**Authors:** Elise Meyn Ung, Cecilie Birkmose Erichsen, Stig Poulsen, Marianne Engelbrecht Lau, Sebastian Simonsen, Annika Helgadóttir Davidsen

**Affiliations:** 1 Department of Psychology, University of Copenhagen, Øster Farimagsgade 2A, 1353 Copenhagen K, Denmark; 2 Stolpegaard Psychotherapy Centre, Mental Health Services, Capital Region of Denmark, Stolpegaardsvej 20, 2820 Gentofte, Denmark

**Keywords:** Eating disorders, Bulimia nervosa, Binge eating disorder, Eating disorder not otherwise specified, Interpersonal problems, Group psychotherapy, Treatment outcome, Eating disorder examination and inventory of interpersonal problems

## Abstract

**Background:**

Interpersonal problems are thought to play an essential role in the development and maintenance of eating disorders. The aim of the current study was to investigate whether a specific interpersonal profile could be identified in a group of patients diagnosed with Bulimia Nervosa, Binge Eating Disorder, or Eating Disorders Not Otherwise Specified, and to explore if specific types of interpersonal problems were systematically related to treatment outcome in this group of patients.

**Methods:**

The participants were 159 patients who received systemic/narrative outpatient group psychotherapy. Interpersonal problems were measured at baseline, and eating disorder symptoms were measured pre- and post treatment. Data were analysed with the Structural Summary Method, a particular method for the analysis of the Inventory of Interpersonal Problems, and hierarchical regression analysis was conducted.

**Results:**

The patients demonstrated a generally Non-assertive and Friendly-submissive interpersonal style. No significant association between the overall level of interpersonal problems and treatment outcome was identified. However, the results showed a correlation between being cold and hostile and poor treatment outcome, while being domineering showed a trend approaching significance in predicting better treatment outcome.

**Conclusion:**

The results indicate that patients with eating disorders show a specific interpersonal profile, and suggest that particular types of interpersonal problems are associated with treatment outcome.

## Plain English summary

Difficulties in interpersonal relationships are common among people with eating disorders. We investigated the association between interpersonal problems, eating disorders, and the effect of group psychotherapy. The results demonstrated that patients with eating disorders show interpersonal difficulties characterized by being too friendly and non-assertive. Further, we found an association between two specific types of interpersonal problems and the effect of systemic/narrative group psychotherapy: being too cold was associated with poorer treatment outcome, while being more domineering appeared to be related to better treatment outcome. This information, combined with further research, may be useful in planning treatment for patients with eating disorders in the future.

## Background

Eating disorders are a group of common and serious mental disorders, characterised by disturbances in eating patterns with significant psychological, physiological, and social consequences [1]. Interpersonal problems have been suggested to be a core component in the development and maintenance of eating disorders [2, 3]. Interpersonal problems encompass a broad range of issues related to a person’s social functioning and ability to create and maintain healthy and meaningful relationships with significant others [4]. A number of studies have shown that both specific types and the overall level of interpersonal problems at baseline are associated with poorer outcome of treatment for eating disorders [4–7]. A more thorough understanding of this relationship, including identification of interpersonal predictors of outcome, can hopefully be used in the development of interventions tailored to the individual patient, thereby leading to more efficient treatment of eating disorders.

**Fig. 1:**
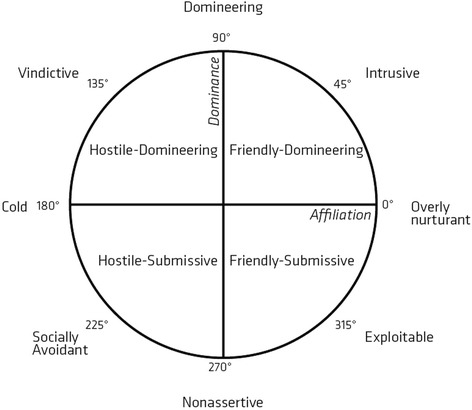
Title: The interpersonal circumplex divived into eight octants and four quadrants

A substantial part of the research regarding the association between interpersonal problems and eating disorders is based on the interpersonal circumplex and the Inventory of Interpersonal Problems (IIP) developed by Horowitz et al. [8]. IIP is based on a two-dimensional circumplex model in which all interpersonal behaviours can be classified based on the specific behaviour’s unique combination of the two underlying dimensions: *affiliation* and *dominance*. According to interpersonal theory, these dimensions are essential elements in interpersonal behaviour [9–11]. The interpersonal circumplex can be divided into four quadrants and eight octants, as illustrated in Fig. 1.

A range of empirical studies has implied that IIP is a valid instrument for predicting therapeutic outcome across various diagnoses and treatment methods [12–21].

Several studies indicate that being hostile and hostile-dominant are associated with poorer treatment outcome, while being friendly and friendly-submissive are related to better outcome [12–18]. Other studies have found that specific types of interpersonal problems are not related to treatment outcome [19–21]. In addition, the results concerning the correlation between the overall level of interpersonal problems and treatment outcome are contradictory. Some studies find that a high level of interpersonal problems is related to poorer outcome [21–23], while others find that such a level predicts better outcome [14].

Previous research has suggested that eating disorders, regardless of the specific eating disorder diagnosis, are associated with a friendly-submissive and non-assertive interpersonal profile, which indicates a lack of confidence and self-esteem, and difficulties with being assertive [5, 6]. Studies of interpersonal predictors of treatment outcome among patients with eating disorders have shown mixed results. A number of studies have found significant associations between an elevated level of the overall IIP-score and poorer treatment outcome [6, 24, 25]. More specifically, problems in the octants *Domineering* and *Socially avoidant* have been found to negatively predict treatment outcome [5, 6]. Conversely, other studies have found that IIP-profiles do not predict treatment effect [26–28]. The results from these studies are difficult to generalize due to the diversity of treatment modalities, including combinations of individual and group psychotherapy.

Since only relatively few studies of IIP as a predictor of outcome in treatment for eating disorders exist and the results of these studies are conflicting, more research is needed before IIP-data can be used in the planning of clinical practice for patients with eating disorders. More specifically, to the best of our knowledge no prior studies have examined the association between specific types of interpersonal problems and treatment outcome in patients with eating disorders treated with systemic/narrative group psychotherapy. Accordingly, the aim of the current study was to explore the association between specific types and/or level of interpersonal problems and treatment outcome of systemic/narrative group psychotherapy in patients diagnosed with Binge Eating Disorder (BED), Bulimia Nervosa (BN), or Eating Disorders Not Otherwise Specified (EDNOS). Furthermore, the purpose of the study was to investigate if the three diagnostic groups (BN, BED, and ED-NOS) showed similar or different interpersonal profiles.

## Methods

### Participants

The patients in the study were treatment-seeking adults referred to outpatient treatment at Stolpegaard Psychotherapy Centre (SPC), Mental Health Services in the Capital Region of Denmark, and participating in the randomized controlled trial, F-EAT: *Feedback* versus *no feedback in improving patient outcome in group psychotherapy for eating disorders* [29, 30]. Criteria for inclusion in the trial were: age of 18 years or older, diagnosis of BED, BN, or EDNOS according to DSM-IV, body mass index (BMI = kg/m^2^) ≥ 20, and written consent. Exclusion criteria were: acute suicidal risk, psychosis, severe depression, alcohol abuse, intake of medicine and/or illegal substances up to three months before referral, severe or non-regulated physical co-morbidity, pregnancy, inability to understand Danish, previous participation in the trial, concurrent psychotherapeutic/psychiatric treatment outside SPC, lack of informed consent, or considered unable to attend treatment sessions as planned.

A total of 159 patients participated in the F-EAT trial, which investigated if the use of Feedback Informed Treatment (FIT) could reduce dropout and improve treatment outcome for patients with eating disorders. Neither drop-out rates nor differences between the EDE Global scores of the two treatment groups were significant at baseline nor after treatment [30]. Data were therefore pooled to one group in the present study.

### Treatment

SPC offers outpatient treatment to adults with non-psychotic psychiatric disorders [31]. The treatment of eating disorders is primarily group psychotherapy combined with consultations with a dietician, physiotherapist, consulting physician, and social worker. Every group consists of 7 patients and 2 therapists. The group meets once a week for 2.5 h, and works as a rolling group where new patients start as others end treatment. The length of the treatment course is 20 sessions for patients with BN and EDNOS, and 25 sessions for patients with BED. Patients are weighed before each session and are encouraged to set individual goals for treatment [32].

The group therapy is based on systemic/narrative theory and a post-modern view on patients and therapy [33, 34], where patients are considered as experts on their own life [32]. Since an essential focus in narrative therapy is the creative character of the language, the use of verbal externalization of the eating disorder is an important part of the treatment [35, 36]. The group treatment is best described as individual treatment in the group, where each patient is interviewed at every group session. The remaining participants in the group are included as a reflecting team [37]. A substantial part of the treatment is participants being asked to keep a food diary, which is then discussed in the group sessions.

### Measures

#### Baseline measures

Socio-demographic and diagnostic information (age, gender, diagnosis, duration of eating disorder, BMI, children, marital status, education, employment status, comorbidity, and EDE) were collected together with IIP at baseline.

#### Inventory of interpersonal problems (IIP-C)

IIP-C is a self-report instrument consisting of 64 items that measure interpersonal difficulties on the dimensions *control* and *affiliation* [38]. The two dimensions divide the two-dimensional space in four quadrants: Friendly-Dominant, Hostile-Dominant, Friendly-Submissive and Hostile-Submissive. The instrument consists of eight subscales, each containing eight items that starts with either *“It’s hard for me to…”* or *“I do too much...”.* Respondents must rate their level of distress for each item. The rating is done on a Likert scale ranging from 0 (not at all) to 4 (extremely) [38]. IIP-C scores are calculated as means on eight scales corresponding to the eight octants in the circumplex model (*Domineering, Vindictive, Cold, Socially Avoidant, Non-assertive, Exploitable, Overly-nurturant, and Intrusive)* and a total scale. IIP has shown acceptable internal consistency, construct validity, and criterion validity. The test-retest reliability is good for the total scale [8].

#### Eating disorder examination (EDE 12.0)

EDE 12.0 is a semi-structured interview which is intended to provide a detailed picture of the specific psychopathology in patients with eating disorders [39]. The interview focuses primarily on the previous 28 days and consists of 15 diagnostic questions and 23 scale-questions. EDE has four subscales: Shape concern, Weight concern, Eating concern, and Dietary restraint, which can be combined in a global score that reflects the total mean of the four subscales. A high EDE Global score indicates a high level of eating disorder symptoms and distress.

EDE has shown satisfactory psychometric properties in the form of high inter-rater reliability, test-rest reliability above 0.7, and good discriminant validity and internal consistency [40–42].

### Outcome measures

#### Eating disorder examination (EDE 12.0)

EDE is the golden standard for outcome measures in eating disorders, and the EDE Global score is therefore used as the outcome measure in this study.

### Data analysis

ANOVA analyses were conducted to explore if differences in IIP total and subscale scores between the three diagnostic groups could be identified at baseline. Post hoc analyses revealed where potential differences could be found and gave rise to the execution of individual t-tests. These analyses, together with the subsequent ANOVA, t-test, and hierarchical regression analyses, were conducted using SPSS version 24.

To categorize patients according to their interpersonal profile, we used Structural Summary Method (SSM), as recommended by Gurtman & Balakrishnan [43]. SSM calculates the *prototypicality* of a group or an individual, i.e. can be described with a value of R^2^ between 0 and 1, which indicates the extent to which the profile corresponds to the theoretically expected correlations in the circumplex model, and if data can be described with a sinusoidal formula and curve. SSM consists of three structural parameters: *Elevation* (the general level of interpersonal distress), *Amplitude* (the degree of differentiation of the profile), and *Angular displacement* (a measure of interpersonal theme or typology). These three parameters can only be interpreted as providing relevant information about the interpersonal profile of a group or an individual in the case of high prototypicality (>0.8).

Hierarchical multiple regression analyses, with the use of three blocks, were conducted to examine if IIP subscales significantly predicted treatment outcome. The dependent variable was EDE Global score post-treatment. In block 1, the eight IIP-scales were added as predictors. To control for the effect of EDE Global baseline and diagnoses, these variables were added as covariates in block 1. In block 1, the method ENTER was used, and the three diagnoses were dummy coded. In block 2, the diagnosis variables were removed. By examining the value of the R^2^ change after removing the diagnosis variables, it was determined whether distinguishing between diagnoses significantly contributed to the explanatory value of the model. A backward elimination procedure was conducted in block 3 to exclude non-significant predictors in a stepwise manner. In a backward elimination procedure, all variables are initially added to the model simultaneously, and the contribution of each variable to the model is determined. A predictor is thereafter removed if it does not significantly contribute to the explanatory value of the model. Finally, the model will only include the predictors that significantly contribute to the explanation of the outcome variable. The same procedure was conducted in an analysis where the only predictor variable was IIP-total.

Prior to the statistical analyses, assumptions regarding normal distribution were tested and found to be met.

## Results

### Sample characteristics

The majority of the patients were single female students without children. BN was the most frequent diagnosis in the sample. See Table 1 for further baseline data.

**Table 1:** Baseline characteristics for patients. *N* = 159

Socio-demographic data		
Age in years, mean, (SD)	26.9	(8.7)
Female, n (%)	156	(98.1)
Marital status: single, n (%)	112	(70.4)
Children under the age of 15: no, n (%)	133	(83.6)
Education: ≥ 10 years of schooling, n (%)	125	(78.6)
Employment status: student, n (%)	71	(44.7)
Diagnostic data		
BN, n (%)	73	(45.9)
BED, n (%)	29	(18.2)
EDNOS, n (%)	57	(35.8)
Duration of eating disorder >5 år, n (%)	104	(65.4)
Body Mass Index, mean (SD)	26.3	(7.7)
Comorbidity^1^: ≥ 1 comorbid diagnosis, n (%)	48	(30.2)
EDE Global, baseline, mean (%)	3.94	(0.97)
EDE Global, post, mean (%)	2.00	(1.42)

^1^Comorbidity was assessed with Mini International Neuropsychiatric Interview (MINI)

Modified table from Davidsen et al. (2017) [30]

### Interpersonal profiles

Only 150 participants filled out IIP at baseline. Table 2 shows the means and standard deviations of the IIP baseline scores and the eating disorder diagnosis for these 150 patients.

**Table 2:** IIP subscales scores at baseline

Baseline IIP Mean (SD)	Total sample *N = 150*	BN *N = 67*	EDNOS *N = 55*	BED *N = 28*
Domineering	0.61 (0.54)	0.60 (0.56)	0.65 (0.54)	0.59 (0.46)
Vindictive	0.76 (0.60)	0.72 (0.64)	0.90 (0.61)*	0.57 (0.43)*
Cold	1.06 (0.71)	1.14 (0.82)	1.06 (0.61)	0.87 (0.55)
Socially Avoidant	1.54 (0.86)	1.48 (0.90)	1.59 (0.80)	1.55 (0.86)
Nonassertive	1.85 (0.92)	1.80 (0.99)	1.99 (0.90)	1.72 (0.77)
Exploitable	1.72 (0.84)	1.62 (0.84)	1.84 (0.84)	1.72 (0.84)
Overly-nurturant	1.78 (0.85)	1.64 (0.73)	1.87 (0.97)	1.91 (0.88)
Intrusive	1.19 (0.75)	1.04 (0.76)	1.30 (0.70)	1.30 (0.77)
Total scale	1.31 (0.55)	1.26 (0.56)	1.40 (0.55)	1.28 (0.52)

*t_(81)_ = 2.540, *p* = 0.013(EDNOS:BED)

No significant differences in interpersonal profiles across the three diagnoses were found at baseline (all *p* > 0.1), with the exception of the scale Vindictive, where patients with EDNOS reported significantly higher scores than patients with BED. No significant differences in IIP total scores between the three diagnoses were found (F_(2)_ = 1.106, *p* = 0.333).

As shown in Table 3, the SSM analysis showed high prototypicality for both the entire sample and for each separate diagnosis. Thus, the data are consistent with the expected pattern of correlations and follow a sinusoidal formula [44]. Consequently, the high level of prototypicality allows an interpretation of the structural parameters in Table 3. The total sample showed a friendly-submissive interpersonal style (Angular displacement: 297.94°), with higher levels of interpersonal problems in the octants Non-assertive, Exploitable, and Overly-nurturant.

**Table 3:** SSM data for the total sample and for each eating disorder diagnosis

Structural Summary Method	Total sample *N = 150*	BN *N = 67*	EDNOS *N = 55*	BED *N = 28*
Elevation	1.31	1.26	1.4	1.28
Amplitude	0.61	0.56	0.64	0.68
Angular displacement (°)	297.94	291.1	299.85	308.4
Prototypicality (R^2^)	0.94	0.95	0.94	0.92

Based on the high level of prototypicality of the diagnoses and the total sample, it is assumed that there is no indication of the existence of meaningful subgroups in the sample [45].

### Interpersonal problems as predictors of treatment outcome

Overall, the treatment was effective in reducing eating disorder symptoms, measured with EDE Global, in the total sample [30].

An ANOVA conducted in the current study showed no differences in the treatment effect between the three diagnoses, since the effect*diagnosis interaction was insignificant (F_(2)_ = 0.142, *p* = 0.868). It is worth mentioning though, that the uneven distribution of the three diagnoses might have influenced this result.

A hierarchical multiple regression analysis with backward elimination, controlling for the effect of diagnoses and EDE Global baseline, showed that two IIP subscales were systematically associated with treatment outcome. The two predictors Domineering and Cold were the only subscales that remained in the model through the elimination process, and were therefore the only two predictors that systematically contributed to the model’s explanatory value.

The dummy variables for the diagnoses could be removed from the regression model without any significant changes in R^2^. Diagnosis did not significantly contribute to the model’s explanatory value, and did not influence the outcome.

Table 4 shows the parameters in the final regression model. Baseline scores for EDE Global was the best predictor for treatment outcome; higher EDE Global baseline predicted poorer outcome in the form of higher EDE Global post-treatment score. In addition, the final model shows a positive relationship between the subscale Cold and EDE Global post-treatment. This implies that a higher score on the subscale Cold prior to treatment is significantly related to poorer treatment outcome (a high EDE Global score indicates a high level of problems related to eating disorders). Furthermore, the model has retained the negative predictor Domineering, even though it only shows a trend approaching significance. This might indicate an association between a higher score on the Domineering subscale at baseline, and a better treatment outcome in terms of lower EDE Global score at the end of treatment.

**Table 4:** Hierarchical regression analysis for variables predicting EDE-global score (*N* **=** 100)

Step 8	B	SE	Standardized Beta	*T*	*p*
Constant	−0.73	0.52		−1.41	0.163
EDE Global baseline	0–61	0.13	0.41	4.61	0.000
IIP: Domineering	−0.45	0.24	−0.18	−1.89	0.062
IIP: Cold	0.59	0.19	0.31	3.08	0.003

Dependent variable: EDE Global post-treatment

Baseline symptoms and the two remaining IIP scales explained 32.3% of the total variance in EDE Global post-treatment. A secondary analysis, identical with the first regression analysis except for the fact that the eight IIP subscales were replaced with IIP total as predictor, showed that IIP total did not significantly predict outcome since it was excluded from the model in the backward elimination process.

## Discussion

The primary aim of this study was to investigate the association between interpersonal problems and eating disorders, and to determine if specific types of interpersonal problems were systematically related to treatment outcome for this group of patients. To our knowledge, the current study is the first to investigate this exact association in patients with eating disorders treated with systemic/narrative group therapy.

In accordance with previous research [5, 6], our results showed that the group of patients generally showed a friendly-submissive interpersonal style since they reported higher levels of interpersonal problems in the octants Non-assertive, Exploitable, and Overly-nurturant than the rest of the octants. This pattern of interpersonal problems was identified across the three diagnoses in the sample; EDNOS, BED, and BN. Through the course of treatment, the patients’ eating disorder symptoms changed, and they reported a significantly lower level of symptoms at the end of treatment compared to baseline [30]. The treatment effect was not dependent on diagnosis.

The fact that we did not find any differences between the diagnostic groups in regard to interpersonal profile or treatment outcome, can partly be interpreted as support for Fairburn’s transdiagnostic model, in which the classical DSM-categorization of eating disorders is re-evaluated, and the different diagnoses are replaced by the term “eating-disorders” [46, 47]. If the categorization of eating disorders into different diagnoses would be meaningful, one would expect the subgroups to differ on other variables than eating disorder symptoms, such as treatment outcome and interpersonal problems [48]. Our results, indicating no differences between diagnoses in terms of treatment outcome and interpersonal problems, thus tend to support a transdiagnostic understanding of eating disorders.

Hierarchical regression analyses revealed that two IIP subscales, Domineering and Cold, were systematically correlated to outcome, while the overall level of interpersonal problems did not significantly predict treatment outcome. The absence of a significant relationship between IIP total and outcome in patients with eating disorders is in accordance with the findings of Constatino and colleagues [26], while other studies have found that IIP-total *is* associated with treatment outcome [6, 24, 25]. With regard to the finding that higher levels of interpersonal problems of the Cold type significantly predicted poorer treatment outcome for the patients (*p* = 0.003), previous research, investigating a broad spectrum of mental disorders treated with different therapeutic methods, has indicated that interpersonal problems in the quadrant Hostile-Dominant negatively affects treatment outcome [6, 12–17, 49–51]. Of these, only one study exclusively deals with the effect of group therapy [14], and another study specifically investigates eating disorders [6]. Since the octant Cold is partly located in the quadrant Hostile-Dominant, the present results appear to be in accordance with this research.

A high score on the IIP subscale Cold indicates a poor ability to express emotions towards others and difficulties in participating in and maintaining long-lasting relationships [38]. A person who scores high on this scale describes him- or herself as a “lone wolf” that enjoys freedom from social demands. The fact that a Cold interpersonal style negatively predicts treatment outcome for group therapy intuitively appears clinically meaningful since this interpersonal style conflicts with participation and engagement in group therapy. This result is fairly intuitive, and could be expected to be true across many different diagnostic groups. Group therapy demands motivation and ability to express and share emotions and thoughts with others, and to participate in the relational interactions in the group [52], which is incongruent with the description of the subscale Cold. To the extent that this result is valid for other samples of patients with eating disorders, it might indicate that patients diagnosed with an eating disorder, who report a high level of baseline scores in the Cold octant, may profit more from other types of treatment than group therapy.

A high score on the subscale Domineering indicates problems related to control and manipulation. In the present study, this subscale showed a trend approaching significance in predicting better treatment outcome (*p* = 0.062). However, the finding is contrary to previous research, which generally finds that a domineering interpersonal style is negatively associated with treatment outcome. One explanation of this apparent contradiction may be that the subscale Domineering is the subscale in which the patients in the sample have the *lowest* scores. Thus, the fact that a higher score on this subscale appears to be related to better outcome in the present study, may be a consequence of the particularly low baseline score found in the sample on this scale. In other words, our results may indicate that a certain level on the scale Domineering is advantageous for the therapeutic outcome, while a higher, more pathological level, is unfavourable. It should be noted that since there is only a trend towards a significant relationship, these considerations are only preliminary, and further research is needed.

Previous research using the IIP includes different diagnostic groups and diverse forms of treatment, which makes generalizations and recommendations for clinical practice difficult. If measures of interpersonal problems are to have implications for clinical practice within the field of eating disorders, further research focusing exclusively on eating disorders treated with a variety of specific treatment methods is needed. The knowledge generated by such studies may be used in the development of more effective, specifically tailored treatments for patients with eating disorders.

The present study has certain limitations. First, all patients were recruited from the same treatment institution, which is specialized in outpatient treatment. The fact that in-patients or patients in day treatment are not included in the sample creates a potential selection bias, precluding the generalization of the results to patients with more severe eating disorders. It may also be discussed whether the inclusion of patients with BED and BN is optimal from a clinical perspective since these two groups tend to differ in demographic variables such as age and gender. Secondly, the fact that IIP relies on self-report may be a source of bias. It is possible and even likely that the patient’s view of their own interpersonal difficulties is different from other people’s view of the patient’s difficulties. To meet this challenge, it would be relevant to include the view of significant others in the assessment of the patient’s interpersonal problems [6]. A third limitation in the present study is that we do not have the opportunity to compare our sample to a control group, since the Danish reference sample is not publicly available. Fourth, It may be argued that due to multiple testing the significance levels should have been reduced in the ANOVA analysis. Fifth, we used EDE Global to measure eating disorder severity. This measure does not necessarily reflect frequency of binging, which is a core symptom of both BED and BN. Finally, it may be problematized that our sample exclusively includes patients diagnosed with BN, BED, and EDNOS, while patients with Anorexia Nervosa are not represented in the study (they were excluded from the original RCT, as they received a different kind of treatment). Thus, the implications of the present study for the general clinical practice with patients with eating disorders are limited.

## Conclusion

The purpose of the current study was to investigate interpersonal problems in a group of patients with BN, BED, and EDNOS, and to assess if these were systematically related to treatment outcome in systemic/narrative group therapy. We found that patients predominantly experience interpersonal problems in the octants Nonassertive, Exploitable and Overly-nurturant. Furthermore, a higher score on the subscale Cold was identified as a significant predictor of poorer outcome, while the subscale Domineering showed a trend approaching significance in predicting better treatment outcome. No significant association between the overall level of interpersonal problems and treatment outcome was identified. Previous research in this particular area is sparse, and more research is therefore needed to further illuminate the relationship between interpersonal problems and outcome for patients with eating disorders.

## Abbreviations

BED: Binge Eating Disorder
BN: Bulimia Nervosa
DSM IV: Diagnostic Statistical Manual
EDNOS: Eating Disorder Not Otherwise Specified
SSM: Structural Summary Method
